# Quality Studies on *Cynometra iripa* Leaf and Bark as Herbal Medicines

**DOI:** 10.3390/molecules29112629

**Published:** 2024-06-03

**Authors:** Shabnam Sabiha, Kamrul Hasan, Katelene Lima, Maryam Malmir, Rita Serrano, Isabel Moreira da Silva, João Rocha, Nurul Islam, Olga Silva

**Affiliations:** 1 Research Institute for Medicines (iMed.ULisboa), Faculty of Pharmacy, Universidade de Lisboa, 1649-003 Lisbon, Portugal; s.sabiha@edu.ulisboa.pt (S.S.); mdkamrul@edu.ulisboa.pt (K.H.); k.lima@edu.ulisboa.pt (K.L.); m.malmir@edu.ulisboa.pt (M.M.); rserrano@edu.ulisboa.pt (R.S.); isabelsilva@edu.ulisboa.pt (I.M.d.S.); jrocha@ff.ulisboa.pt (J.R.); 2 Department of Zoology, Faculty of Biological Sciences, University of Rajshahi, Rajshahi 6205, Bangladesh; n_islamm@yahoo.com

**Keywords:** antioxidant, *Cynometra iripa*, chemical profile, herbal substance, light microscopy, macroscopic analysis, mangrove species, quality control

## Abstract

*Cynometra iripa* Kostel. is a *Fabaceae* species of mangrove used in traditional Ayurvedic medicine for treating inflammatory conditions. The present study aims to establish monographic botanical and chemical quality criteria for *C. iripa* leaf and bark as herbal substances and to evaluate their in vitro antioxidant potential. Macroscopic and microscopic qualitative and quantitative analyses, chemical LC-UV/DAD-ESI/MS profiling, and the quantification of key chemical classes were performed. Antioxidant activity was evaluated by DPPH and FRAP assays. Macroscopically, the leaf is asymmetrical with an emarginated apex and cuneate base. Microscopically, it shows features such as two-layered adaxial palisade parenchyma, vascular bundles surrounded by 3–6 layers of sclerenchyma, prismatic calcium oxalate crystals (5.89 ± 1.32 μm) along the fibers, paracytic stomata only on the abaxial epidermis (stomatal index–20.15), and non-glandular trichomes only on petiolules. The microscopic features of the bark include a broad cortex with large lignified sclereids, prismatic calcium oxalate crystals (8.24 ± 1.57 μm), and secondary phloem with distinct 2–5 seriated medullary rays without crystals. Chemical profile analysis revealed that phenolic derivatives, mainly condensed tannins and flavonoids, are the main classes identified. A total of 22 marker compounds were tentatively identified in both plant parts. The major compounds identified in the leaf were quercetin-3-*O*-glucoside and taxifolin pentoside and in the bark were B-type dimeric proanthocyanidins and taxifolin 3-*O*-rhamnoside. The total phenolics content was higher in the leaf (1521 ± 4.71 mg GAE/g dry weight), while the total flavonoids and condensed tannins content were higher in the bark (82 ± 0.58 mg CE/g and 1021 ± 5.51 mg CCE/g dry weight, respectively). A total of 70% of the hydroethanolic extracts of leaf and bark showed higher antioxidant activity than the ascorbic acid and concentration-dependent scavenging activity in the DPPH assay (IC_50_ 23.95 ± 0.93 and 23.63 ± 1.37 µg/mL, respectively). A positive and statistically significant (*p* < 0.05) correlation between the phenol content and antioxidant activity was found. The results obtained will provide important clues for the quality control criteria of *C. iripa* leaf and bark, as well as for the knowledge of their pharmacological potential as possible anti-inflammatory agents with antioxidant activity.

## 1. Introduction

*Cynometra iripa* Kostel., commonly known as “Shingra”, is classified as “Least Concern” in the IUCN Red List of Threatened Species [1]. It is a globally recognized mangrove species [2,3] that belongs to the *Fabaceae* (*Leguminosae*) family and to the polyphyletic *Cynometra* L. genus, which comprises 113 species of shrubs to large trees [4,5,6,7,8,9,10]. *C. iripa* is a characteristic species of mangrove swamps [11], exhibiting a scattered distribution. It is present in various regions, including India, Bangladesh, Myanmar, Thailand, Northeast Australia, Papua New Guinea, Eastern Indonesia (such as West Irian, Halmahera, Moluccas, Seram, Ambon, Aru, and Tanimbar Islands), and the Philippines, ranging from Panay Island to Mindanao [1].

**Figure 1:**
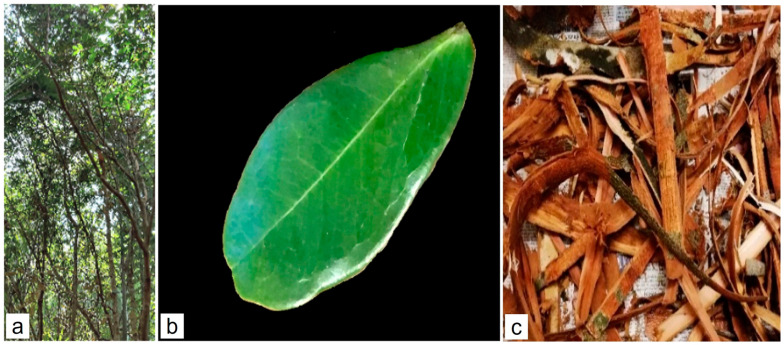
*Cynometra iripa* (**a**) general aspect; (**b**) fresh green leaf; (**c**) fresh bark.

*C. iripa* is a small tree (6–15 m), sometimes multi-stemmed (Figure 1). The leaf (Figure 1a) is green color, 1–2-jugate, and asymmetrical. The flower is aromatic and appears in white or a delicate pale pink hue. The fruit is one-seeded, asymmetrical, with a pronounced beak at the apex of the dorsal suture, extending partially along the dorsal side. It is suborbicular, laterally compressed, deeply wrinkled, woody, and transitions from green to brown as it matures. The bark (Figure 1b) is smooth, displays brown-grey and patchy tones, and is finely fissured [12,13,14].

Traditionally, in India, a paste of the leaf, seed, and stem of *C. iripa* is used to heal wounds [15], and a decoction of the leaf is used to treat ulcers [16]. Tribal people extract oil from the seeds to treat cholera [17]. Although this species is found in Bangladesh and traditionally used by local people, no specific therapeutic indications have been found in the literature.

Some chemical studies have already been conducted on different *C. iripa* plant parts (Table 1). A total of 10 fatty acids were detected in the leaf oil, while 14 fatty acids were detected in the seed oil [18]. Fifteen compounds were identified by GC-MS from the seed and seed coats of this species [19,20]. Basak et al. (1996) reported the presence of chlorophyll, carotenoids, proteins, polyphenols, and tannins in the leaf ethanolic extract of this species. The carotenoid, polyphenol, tannin, and protein contents were 0.08, 30.15, 18.34, and 22.58% of the dry weight of the extract, respectively [21].

Methanol, ethyl acetate, and chloroform–methanol (1:1) extracts of *C. iripa* leaf showed antibacterial activity against two strains of *Aeromonas hydrophila*, *Edwardsiella tarda*, *Pseudomonas fluorescens*, *Pseudomonas aeruginosa*, and *Vibrio alginolyticus* by the diffusion method [22]. It has also been reported that an ethanolic and methanolic extract of the *C. iripa* aerial parts showed in vitro antimicrobial activity against *Bacillus cereus*, *P. aeruginosa*, *Staphylococcus aureus*, and *Salmonella typhimurium* through the diffusion method. In comparison to ethanolic extracts, the methanolic extracts of leaf, stem, early seed, mature seed, and seed coat presented higher antimicrobial activity against *P. aerunginosa* [23]. In addition, the methanol extract of the bark showed antifungal activity against *Alternaria alternata* and *Fusarium moniliforme* using the poison food technique [24].

*C. iripa* is often confused with another species, *Cynometra ramiflora* (L.), and previously, it was described as a variety of this species. According to the original description made by Linnaeus (1753), *C. ramiflora* is characterized by unijugate leaves [25], whereas Kosteletzky (1835) described *C. iripa* as having bijugate leaf [26]. For this reason, *C. ramiflora* var. *bijuga* has been considered a synonym of *C. iripa* [11]. Although based on key characteristics like the apex of leaflets, the length of inflorescences, the length of pedicels, the apex of the anthers shape, and the position of the fruit beak, this species is considered to be different [12]. 

As different plant parts of this species are used in Ayurveda and other systems of Indian traditional medicine, to allow their use as herbal medicines, monographic quality parameters are essential, which is the main goal of the present work. Concerning the identification, macroscopic and microscopic analyses of the whole, fragmented, and powdered plant materials (leaf and bark) are performed together with the establishment of the chemical fingerprints and quantification of the main class of compounds. Additionally, in attending to the chemical fingerprint results, the antioxidant potential of the extracts of these medicinal plants is also evaluated. 

## 2. Results

### 2.1. Macroscopic and Microscopic Analyses

#### 2.1.1. Leaf

##### Macroscopic Characteristics

**Figure 2:**
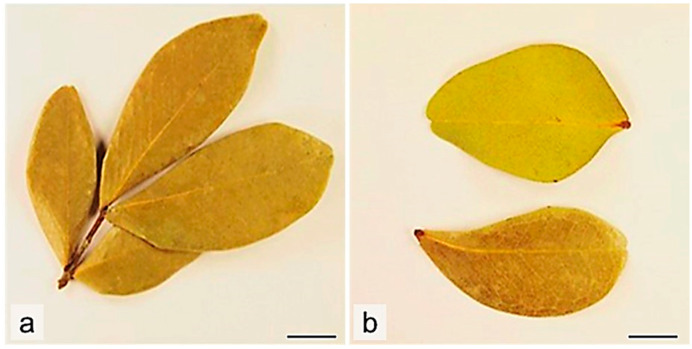
*C. iripa* leaf macroscopic characters. (**a**) dried leaflet; (**b**) abaxial and adaxial view of the asymmetrical leaf. Scale bars: (**a**,**b**) = 1 cm.

**Figure 3:**
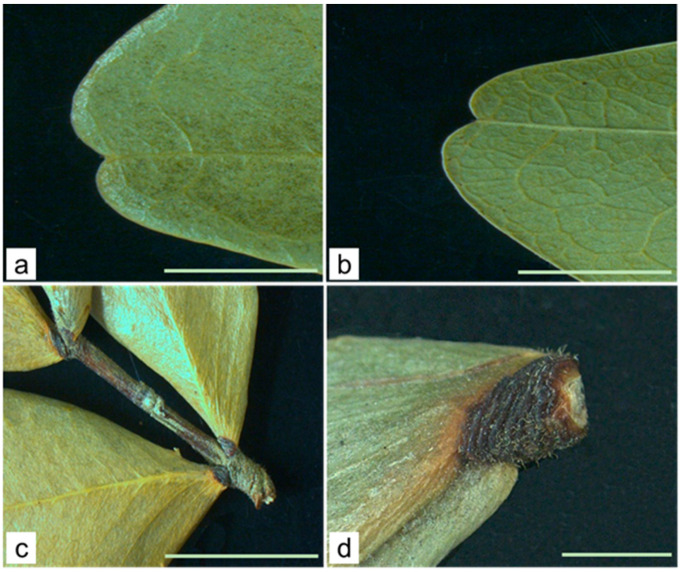
*C. iripa* leaf macroscopic characters. Details of (**a**) adaxial and (**b**) abaxial view of emarginated apex; (**c**) cuneate base, petiolules, and rachis; (**d**) hairy petiolule. Scale bars: (**a**–**c**) = 5 mm; (**d**) = 2 mm.

The macroscopic observation (Figure 2 and Figure 3) revealed a dried grey color asymmetrical leaflet, with leaf lamina 3.0–6.6 cm in length and 1.4–2.8 cm wide, emarginate apex (Figure 3a) and cuneate base (Figure 3c), and venation brochidodromous, prominent on the abaxial surface (Figure 3b) and entire margin. Trichomes were observed on leaf rachis and petiolules (0.2–0.3 cm long) (Figure 3d).

##### Microscopic Characteristics

**Figure 4:**
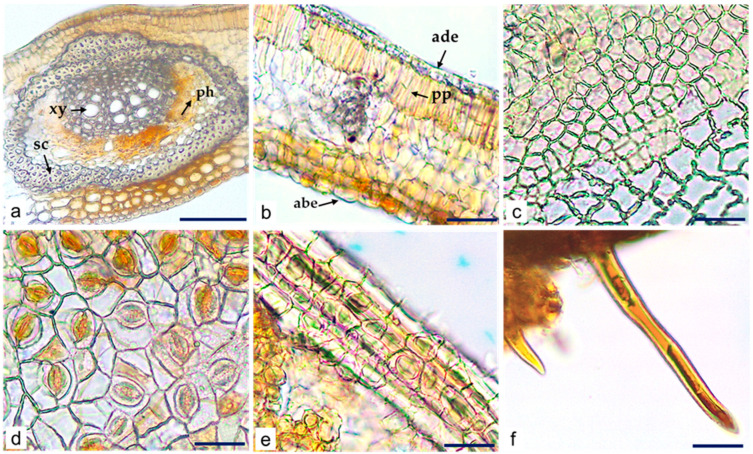
*C. iripa* dried leaf. (**a**,**b**) LM micrographs of transverse section. Details of central vein and mesophyll; (**c**) adaxial surface without stomata; (**d**) abaxial surface with paracytic stomata; (**e**) calcium oxalate prismatic crystals at the vein; (**f**) unicellular trichomes on petiolule; xy—xylem, ph—phloem, sc—sclerenchyma, ade—adaxial epidermis, pp—palisade parenchyma, abe—abaxial epidermis. Scale bars: (**a**) = 100 µm; (**b**–**f**) = 50 µm.

Light microscopy (LM) analysis of transversal sections of *C. iripa* leaf showed rectangular to polygonal upper epidermis cells, smaller (10.26–18.64 µm) than the lower epidermis cells (12.84–34.20 µm), mucilage-containing cells on the abaxial epidermis, and double-layered palisade parenchyma on the adaxial mesophyll tissue. Hypodermal layers were absent. The presence of adaxial xylem and abaxial phloem surrounded by 3–6 layers of sclerenchyma was observed (Figure 4a,b). 

The paracytic type of stomata (two subsidiary cells parallel to the guard cells) was only detected on the abaxial surface (Figure 4c,d). Calcium oxalate prismatic crystals (3.52–8.70 µm) were observed in the veins (Figure 4e), and unicellular and pointed non-glandular trichomes were observed in the petiolules (Figure 4f).

**Figure 5:**
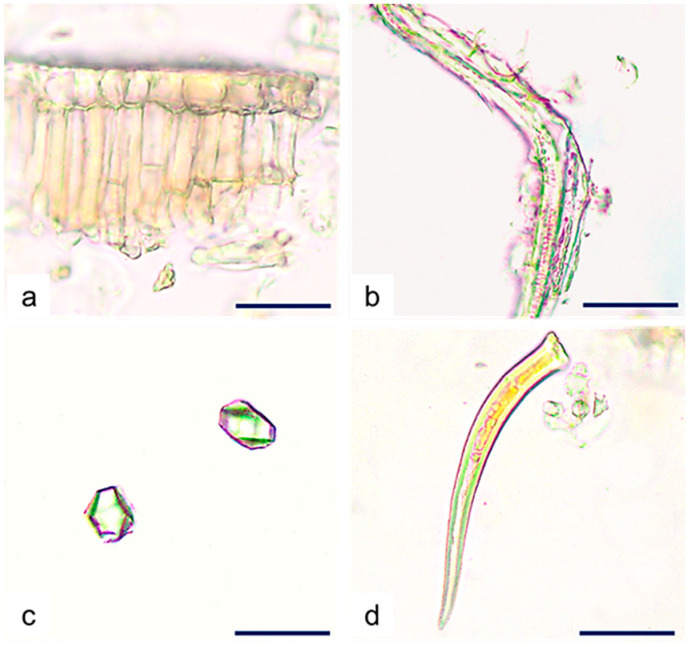
*C. iripa* powdered leaf. Details of (**a**) fragments of epidermal cells and palisade parenchyma; (**b**) fibers; (**c**) free prismatic calcium oxalate crystals; (**d**) unicellular non-glandular trichome. Scale bars: (**a**–**d**) = 50 µm.

The leaf powder of *C. iripa* was greyish-green in color and had a specific odor. By LM, it was possible to identify the presence of characteristic leaf microscopic elements like palisade parenchyma consisting of two layers of cells, fibers, free calcium oxalate prismatic crystals, and free non-glandular trichomes (Figure 5a–d).

#### 2.1.2. Bark 

##### Macroscopic Characteristics

**Figure 6:**
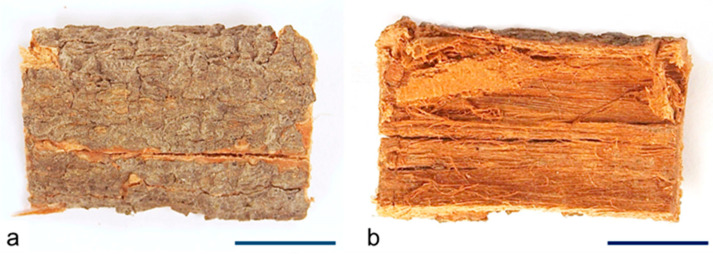
*C. iripa* bark macroscopic characters. Details of (**a**) adaxial view; (**b**) abaxial view. Scale bars: (**a**,**b**) = 1 cm.

The dried stem bark was nearly flat in the piece, smooth, brown-grey in color, and finely fissured; thickness is usually 2–3 mm (Figure 6a,b).

##### Microscopic Characteristics

**Figure 7:**
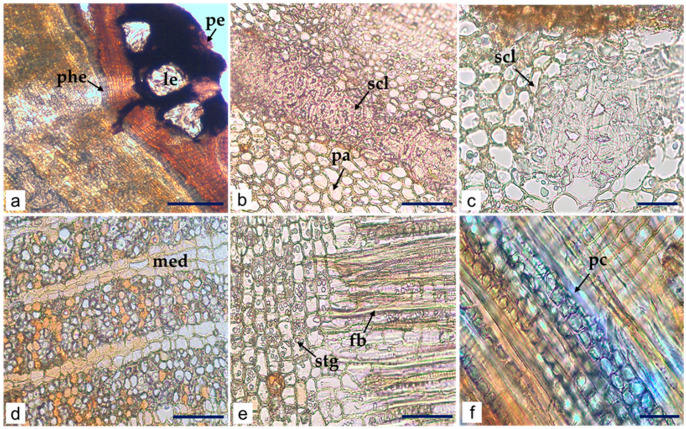
Microscopic characters of *C. iripa* bark. Details of (**a**–**d**) transverse section of bark showing lenticel (le), periderm (pe), phelloderm (phe), and cortex; layer of thick sclereids (scl) and parenchyma cell (pa); a group of sclereids (scl); medullary rays (med) with 2–5 cell layers in secondary phloem; longitudinal sections (**e**,**f**) showing fibers (fb), calcium oxalate prismatic crystals (pc), starch granules (stg), at parenchyma (**e**); calcium oxalate prismatic crystals (pc) associated with fibers (**f**). Scale bars: (**a**) = 200 µm; (**b**,**d**,**e**) = 100 µm; (**c**,**f**) = 50 µm.

LM analysis of the *C. iripa* bark transversal sections showed the presence of lenticel, periderm, narrow phelloderm (composed of tangentially elongated cells), broad cortex with large elliptical groups of sclereids (heterogenous in shape and size), parenchyma (Figure 7a,b), numerous calcium oxalate prismatic crystals (Figure 7c), and secondary phloem with distinct 2–5 seriated medullary rays (Figure 7d). No calcium oxalate prismatic crystals were found on medullary rays (Figure 7d).

The LM longitudinal section analysis revealed the presence of fibers, some with calcium oxalate prismatic crystals along and parenchyma cell layers and numerous irregularly shaped starch granules (1.65–5.96 µm), isolated or conjugated, scattered in cell layers (Figure 7e,f). 

**Figure 8:**
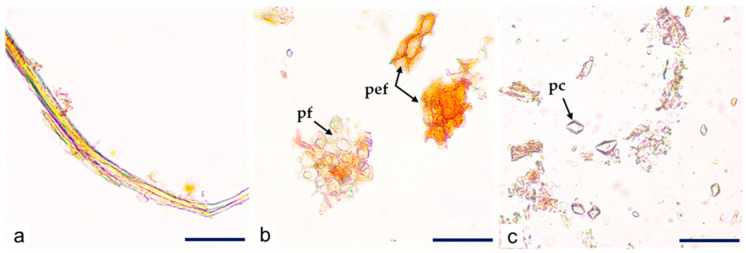
*C. iripa* powdered bark. Details of (**a**) fragments of fibers; (**b**) fragments of parenchyma (pf) and periderm (pef); (**c**) scattered calcium oxalate prismatic crystals (pc); scale bars: (**a**–**c**) = 50 µm.

The powdered *C. iripa* bark was greyish brown in color and characterized by the presence of fragments of fibers (Figure 8a), fragments of parenchyma and reddish-brown periderm (Figure 8b), calcium oxalate prismatic crystals (Figure 8c), and occasional starch granules. 

### 2.2. Quantitative Microscopic Analysis

The principal microscopical characteristics of *C. iripa* leaf and *C. iripa* bark were quantified to provide additional distinctive elements for quality control purposes of these medicinal plants as possible herbal drugs. The results are presented in Table 2. Noticeably, the abaxial epidermal cells were larger in the leaf than the adaxial epidermis cells, and the calcium oxalate prismatic crystals were wider in the bark than in the leaf. 

### 2.3. Chemical Studies

#### 2.3.1. Yield of Extraction

Chemical studies were performed using extracts prepared with botanically characterized raw plant materials. The obtained extraction yields and drug extract ratio (DER) are presented in Table 3. The extract yield percentage was higher in *C. iripa* bark (CIB) than in the *C. iripa* leaf (CIL), corresponding to a lower DER, as verified.

#### 2.3.2. Qualitative Phytochemical Analysis 

A portion of each extract (CIL and CIB) was analyzed using characteristic colorimetric methods for secondary metabolites. The results (Table 4) confirm the absence of alkaloids in both plant extracts, whereas the presence of phenolic compounds (in ferric chloride test, the bluish-black color formation, and in the acetic acid test, the red color formation) and triterpenoids, namely saponins (stable foam formation), is confirmed in both (CIL and CIB) extracts. 

#### 2.3.3. LC-UV/DAD-ESI/MS Fingerprint

**Figure 9:**
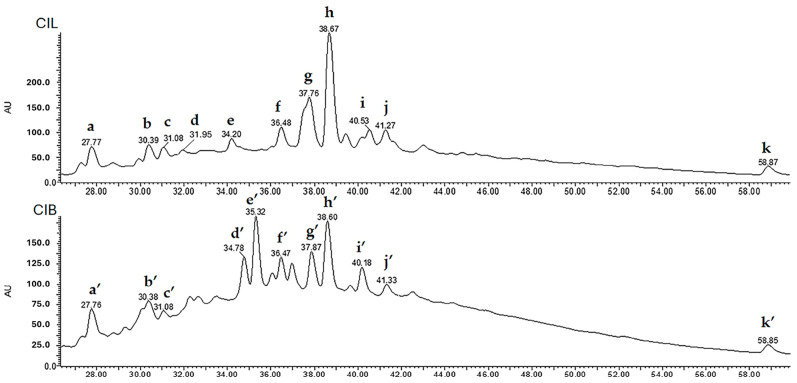
LC/MS chromatographic profile of main marker compounds in *C. iripa* leaf (CIL) and *C. iripa* bark (CIB).

The obtained results of the analysis by high-resolution liquid chromatography coupled to a photodiode array and a mass spectrometry detector using electrospray ionization (LC-UV/DAD-ESI/MS) are presented in Table 5 and Table 6 and Figure 9. The tentative identification of the main compounds was assigned by co-chromatography with authentic standards, comparison of their UV spectra and retention time, and mass spectrometric data based on the PubChem database and different scientific literature. Negative ionization data were selected for identification.

Table 5 presents data on the main compounds identified from *C. iripa* leaf extracts by LC-UV/DAD-ESI/MS. The obtained chromatograms for CIL extracts showed a total of 11 major peaks. Peak **a** showed a [M − H]^−^ ion at *m*/*z* 1154 and fragment ions at *m*/*z* 865 [M − H − 289]^−^, 577 [M − H − 289 − 288]^−^, and 425 [M − H − 289 − 288 − 152 ]^−^ that formed due to RDA fragmentation (one of the most common fragmentation pathways of B-type proanthocyanidins), and 287, that is a monomeric catechin unit, formed due to quinone methide cleavage [27]. Notably, for B-type proanthocyanidins, fragments form the monomeric ions of *m*/*z* 287 or *m*/*z* 289 [27], and according to Karonen et al., 2004, B-type procyanidin oligomers are composed of multiple monomer subunits with interflavonoid C-C linkages that differ by multiples of 288 [28]. Considering the differences between monomer units, UV spectra, and fragmentation pattern, this compound was identified as a B-type proanthocyanidins tetramer. 

Peak **b** exhibited a [M − H]^−^ ion at *m*/*z* 865 and fragment ions at *m*/*z* 577 [M − H − 288]^−^, and 289 (monomeric catechin unit) was identified as a B-type proanthocyanidins trimer. Peaks **c** and **d** showed the [M − H]^−^ ion at *m*/*z* 1442 and subsequent fragment ions at *m*/*z* 1154 [M − H − 288]^−^, 865 [M − H − 288 − 289]^−^, 577 [M − H − 288 − 289 − 288]^−^, and 289 (monomeric catechin unit), which indicated a molecular weight of 1443. Based on the differences between monomer units, UV spectra, and fragmentation pattern, these compounds were also identified as B-type proanthocyanidin pentamers. 

Peaks **e, f,** and **h** presented a [M − H]^−^ ion at *m*/*z* 435, corresponding to a molecular weight of 436, and produced taxifolin aglycone fragment ions at *m*/*z* 303 [M − H − 132]^−^ (indicating loss of a pentose moiety), 285 [M − H − 132 − 18]^−^ (indicating loss of water), and 151 (a fragment produced due to a RDA reaction). The pentose moiety could be attributed to arabinose or xylose. Since arabinose and xylose are monosaccharides with the same molecular formula (C_5_ H_10_ O_5_) and molecular weight (150 g/mol), more experiments are needed to obtain the correct identity of the sugar moiety in these peaks and based on the UV–Vis and MS spectral data, these peaks have tentatively been identified as taxifolin pentoside isomers [29,30]. 

Peak **g** showed a [M − H]^−^ ion at *m*/*z* 463 corresponding to molecular weight 464, with respective fragment ions at *m*/*z* 435 [M − H − 28]^−^, indicating loss of CO, and 301 [M − H − 162]^−^, indicating loss of a glycosyl unit. Based on the UV, fragmentation pattern, and co-chromatography with standards (quercetin-3-*O*-glucoside), this compound was assigned as quercetin-3-*O*-glucoside [31,32].

Peak **i** showed [M − H]^−^ ion at *m*/*z* 433 and characteristic fragment ion at *m*/*z* 301 [M − H − 132]^−^. By comparison of its fragmentation behavior with previous work in the literature, this peak was tentatively identified as quercetin 7-*O*-pentose/apiose [33]. 

Peak **j** showed an *m*/*z* 447 [M − H]^−^ and fragment ions at *m*/*z* 419 [M − H − 28]^−^, indicating loss of CO, and 285 [M − H − 162]^−^, a Kaempferol aglycone formed by the loss of the glycosyl unit and a UV spectrum compatible with its flavonol nature, namely kaempferol-7-*O*-glucoside (MW 448 g/mol). This identity was confirmed by the spectral information of the kaempferol 7-*O*-glucoside of the PubChem database [34].

Peak **k** showed a base peak at *m*/*z* 269 [M − H]^−^ with fragment ions at *m*/*z* 89 and a UV spectrum compatible with its flavone nature, namely apigenin (MW 270 g/mol). This identity was also confirmed by LC/UV-DAD co-chromatography with authentic standards (apigenin).

Table 6 presents data on the main compounds identified from *C. iripa* bark extracts by LC-UV/DAD-ESI/MS. The obtained chromatograms for CIB extracts showed a total of **11** major peaks. Both peaks **a′** and **c′** showed a [M − H]^−^ ion at *m*/*z* 1154 and a similar fragmentation pattern corresponding to the B-type proanthocyanidins tetramer that was observed for peak **a** in CIL. Peak **b′** also showed a [M − H]^−^ ion at *m*/*z* at 865 with the fragmentation behavior of the B-type proanthocyanidins trimer, which was also similar to peak **b** in CIL. 

Peak **d′** and **e′** exhibited a [M − H]^−^ ion at *m*/*z* at 561 and fragment ion at *m*/*z* 433 [M-H-126]^−^ which formed due to RDA fragmentation. The other two fragment ions 287 and 273/271 formed due to a quinine methide reaction (QM) that indicated catechin and afzelechin derivatives, respectively. Based on the UV and fragmentation patterns reported in previous work, these peaks were identified as B-type proanthocyanidins dimers [35]. 

Peak **f′** showed a [M − H]^−^ ion at *m*/*z* 565 and fragment ion 301 [M − H − 264]^−^, a typical fragment for quercetin derivatives formed by the loss of two pentose units (132 + 132). Concerning the UV and fragments behavior, this compound was tentatively identified as quercetin-*3-O*-pentosyl-pentoside [36,37]. 

Peak **g′**, **h′**, and **i′** presented a [M − H]^−^ ion at *m*/*z* 449 and characteristic fragment ions at *m*/*z* 303 [M − H − 146]^−^ assigned to [aglycone H]^−^ 285 and 151, which is similar fragmentation behavior to taxifolin. Based on the obtained UV–Vis and MS spectral data, this peak was tentatively identified as a deoxyhexose (rhamnose) of taxifolin, namely as taxifolin 3*-O*-rhamnoside [38].

Peak **j′** exhibited a [M − H]^−^ ion at *m*/*z* 599 and fragment ions at *m*/*z* 447 [M − H − 152]^−^ formed by the removal of the galloyl moiety and the other fragment ion at *m*/*z* 301 [M − H − 298 (152 + 146)]^−^, indicating the removal of the galloyl-rhamnoside moiety. Based on the UV and typical fragmentation behavior, this compound was identified as quercitrin 3″-*O*-gallate [39]. 

Peak **k′** was tentatively identified as apigenin, similar to peak **k** in CIL as it exhibited a base peak at *m*/*z* 269 [M − H]^−^ with fragment ions at *m*/*z* 89 corresponding to a molecular weight of 270.

**Figure 10:**
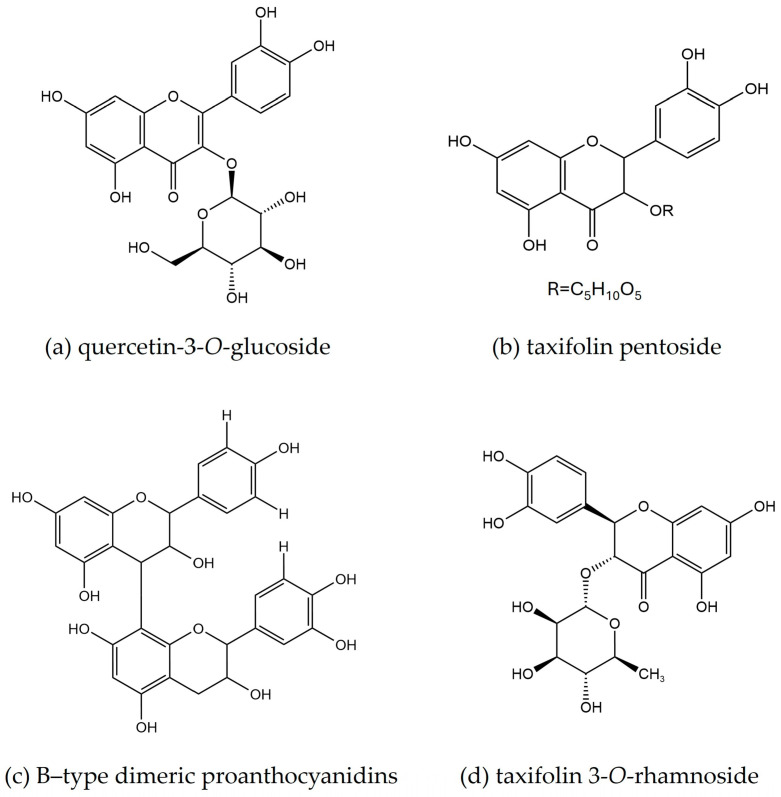
Major identified compounds from leaf (**a**,**b**) and bark (**c**,**d**) hydroethanolic extracts.

Quercetin-3-*O*-glucoside (Figure 10a) and taxifolin pentoside (Figure 10b) were found as the major compounds identified in the CIL, whereas B-type dimeric proanthocyanidins (Figure 10c) and taxifolin 3-*O*-rhamnoside (Figure 10d) were the main compounds identified in the CIB extracts. 

#### 2.3.4. Quantitative Phytochemical Analysis 

From the qualitative analysis, phenolic derivatives were identified as the main chemical class. For this reason, the total phenolic content (TPC), total flavonoid content (TFC), and total condensed tannin content (TCTC) were determined in both extracts and are presented in Table 7. Gallic acid, catechin, and cyanidin chloride were used as standard, respectively. 

TPC was significantly higher (*p* < 0.05) in CIL than CIB, whereas TFC and TCTC were higher (*p* < 0.05) in the CIB extract. 

### 2.4. Antioxidant Activity

#### 2.4.1. DPPH Scavenging Activity

**Figure 11:**
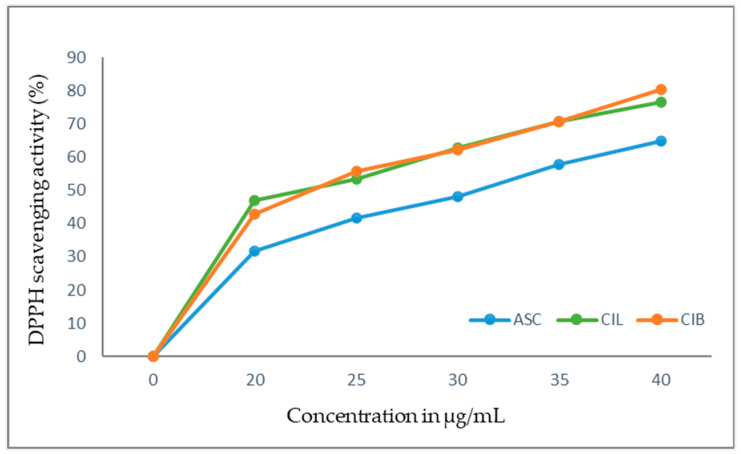
Scavenging activity of *C. iripa* leaf (CIL) and *C. iripa* bark (CIB) extracts; ASC-ascorbic acid.

The results of the scavenging activities of the CIL and CIB extracts by the DPPH method are presented in Figure 11. Both extracts showed a higher antioxidant activity than ascorbic acid (ASC) and concentration-dependent scavenging activity. 

In fact, the IC_50_ (half maximal inhibitory concentration) values of CIL and CIB are similar (23.95 ± 0.93 and 23.63 ± 1.37 µg/mL, respectively) and lower than the obtained value of ASC (30.75 ± 0.51 µg/mL), indicating the higher antioxidant activity of the extracts in comparison with this recognized antioxidant. However, the CIB extract showed the highest percentage of scavenging, 80.3%, at a concentration of 40 µg/mL.

#### 2.4.2. Ferric Reducing Capability

**Figure 12:**
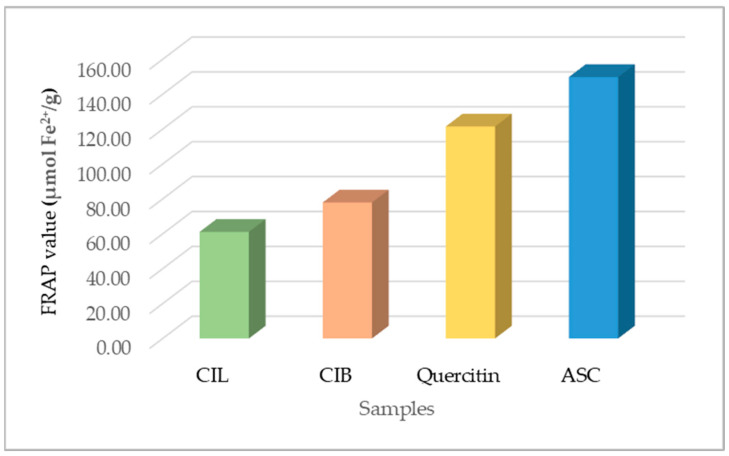
The ferric reducing capacity of *C. iripa* leaf (CIL) and *C. iripa* bark (CIB) extracts; ASC-ascorbic acid.

The results of the ferric-reducing capacity of CIL and CIB determined by the FRAP test are presented in Figure 12. Both the CIL and CIB extracts showed a lower ferric reduction capacity than quercetin and ascorbic acid used as standards. The FRAP value of the CIL extract is 61.11 ± 2.91 µmol Fe^2+^/g dry weight, and of the CIB extract, this is 77.94 ± 2.02 µmol Fe^2+^/g dry weight, while the FRAP value of the quercetin and ASC was 121.51 ± 0.94 µmol Fe^2+^/g dry weight and 149.84 ± 1.08 µmol Fe^2+^/g dry weight, respectively. Comparatively, the CIB extract was shown to have more antioxidant potential than CIL. 

### 2.5. Correlation between Phenolic Content and Antioxidant Activity

The results of the statistical calculation concerning the possible correlation between the phenolic content of the CIL and CIB extracts and the antioxidant potential are presented in Table 8. For both the CIL and CIB extracts, a positive and statistically significant correlation (*p* < 0.05) has been found between the phenolic content and antioxidant activity.

For both the CIL and CIB extracts, TFC and TCTC showed a positive and strong correlation with FRAP activity, giving a Pearson correlation coefficient (r) of 0.99. On the other hand, TPC was positively correlated with DPPH activity, giving an r of 0.85 and 1.00 for CIL and CIB, respectively (Table 8). 

Therefore, the results indicate that different phenolic derivatives, mainly procyanidins, made an outstanding contribution to the antioxidant activity of the CIL and CIB extracts. 

## 3. Discussion 

The quality control of medicinal plant materials is essential to allow them to be used as herbal medicines for human and veterinary use [40]. Therefore, the botanical macroscopic and microscopic characteristics are essential for identifying whole, fragmented, and powdered samples of *C. iripa* leaf and *C. iripa* bark. 

Considering the external leaf morphology observed, *C. iripa* dried leaf has features like alternate jugate leaf arrangement, emarginate apex, cuneate base, and petiolules size up to 0.3 cm similar to those reported by Cooper, W.E. (2015) [13] and Ragavan et al. (2017) [12] for the fresh leaf of this species. In addition, common *Leguminosae* features are noticed for the first time in this species, like paracytic stomata and calcium oxalate prismatic crystals. Saenger and West (2016) referred to the presence of a single palisade layer and the absence of the hypodermal layer as characteristics of *C. iripa* leaf [41]. Our results differ from their study, except for the hypodermal layer, as we found a double layer of palisade parenchyma on the adaxial. Other characteristics found in *C. iripa* leaf are the presence of a vascular bundle surrounded by layers of sclerenchyma, mucilage-filled cells, and paracytic stomata found only on the abaxial surface. Pan (2010) reported the presence of paracytic stomata only on the abaxial surface in another species, *Cynometra chaka*. Furthermore, in *C. chaka* and *Cynometra lujae* De Wild., multicellular uniseriate trichomes were found on the abaxial surface of the leaf [42], whereas in *C. iripa*, non-glandular unicellular trichomes were found only in the petiolule. 

In this study, for the first time, the microscopic features of the dried bark of *C. iripa* are discussed. The most distinctive elements for quality control proposed are the presence of lenticel, periderm, narrow phelloderm, broad cortex with large elliptical groups of sclereids, calcium oxalate prismatic crystals, secondary phloem with 2–5 seriated medullary rays, and irregularly shaped starch granules in all parenchymatous tissues.

The chemical profile analysis showed that phenolic compounds, mainly condensed tannins, and flavonoids, are the main classes identified in *C. iripa* leaf and bark extracts. The major compounds identified in the leaf were quercetin-3-*O*-glucoside and taxifolin pentoside. 

Quercetin and its glycosides are vital plant flavonoids with neuroprotective, cardioprotective, chemo-preventive, antioxidant, anti-inflammatory, and anti-allergic properties [43,44]. These compounds have been shown to suppress inflammatory responses by inhibiting inflammatory enzymes cyclooxygenase (COX) and lipoxygenase [44], and also by inhibiting the production of pro-inflammatory cytokines, such as interleukin-6 (IL-6), tumor necrosis factor-alpha (TNF-α), and interleukin-1 beta (IL-1β), in various cell types [45,46]. Quercetin-3-*O*-glucoside demonstrated strong antioxidant and anti-inflammatory properties in vitro, as it showed the highest activity against cyclooxygenase (COX)-1, COX-2, and lipoxygenase (LOX-5), with IC_50_ values of 3.62, 5.66, and 2.31 µg/mL, respectively. Additionally, it exhibited considerable cytotoxic effects on HeLa cells in a dose- and time-dependent manner [47]. 

Taxifolin is a potent antioxidant that inhibits the increased activity of NF-κB in rats with cerebral ischemia–reperfusion injury [48]. It also exhibited notable anti-inflammatory effects by reducing the transcription of TNF-α, IFN-γ, IL-10, and TLR-4 in Raw 264.7 cells in mice [49]. The other marker compounds detected were B-type trimeric, tetrameric, and pentameric proanthocyanidins, quercetin 7-*O*-pentoside/apioside, kaempferol 7-*O*-glucoside, and apigenin. All these compounds are identified for the first time in *C. iripa* leaf. Different phenolic derivatives are reported to be found in other species of *Cynometra*. For example, proanthocyanidins, taxifolin pentoside, taxifolin 3-*O*-arabinofuranoside, catechin, apigenin 8-*C*-glucoside (vitexin), apigenin 6-*C*-glucoside (isovitexin), kaempferol hexoside, quercetin pentoside, quercetin hexoside, kaempferol–coumaroyl hexoside, isorhamnetin hexoside, and acacetin 7-*O*-*β*-glucoside have been isolated from the ethyl acetate and n-butanol, fractions of the leaf of *Cynometra cauliflora* L. [29,50].

The major compounds detected in the *C. iripa* bark were B-type dimeric proanthocyanidins and taxifolin 3-*O*-rhamnoside. Proanthocyanidins (condensed tannins) are reported to have significant antioxidant, anti-cancer, anti-diabetic, antimicrobial, and immunomodulatory potential [51]. Several studies in the literature reported on the different biological activities of B-type proanthocyanidins like anti-cancer activity by decreasing the in vitro growth of androgen-sensitive (LnCaP) and androgen-resistant (DU145) human prostate cancer cell lines [52], antimicrobial activity against *Candida albicans* and *Cryptococcus neoformans*, with MIC values of 250 to 1000 µg/mL [53], and anti-aging activity by reducing the content of ROS and nicotinamide adenine dinucleotide phosphate oxidases 4 (NOX4) mRNA levels in luteinized granulosa cells (hGC) and tumor granulosa cells (KGN) [54]. 

Taxifolin 3-*O*-rhamnoside is an important flavonoid that showed anti-tumor activity on PANC-1 and A-549 cancer cell lines by inhibiting about 30% of the cell growth at 30 µM concentrations [55]. The other marker compounds detected were B-type trimeric and tetrameric proanthocyanidins, quercetin 3-*O*-pentosyl-pentoside, taxifolin 3-*O*-rhamnoside, quercitrin 3″-*O*-gallate, and apigenin. Like *C. iripa* leaf, all these compounds are identified for the first time in the *C. iripa* bark. There are no other studies in the literature found concerning compounds in the *C. iripa* bark.

The bark extracts of different *Fabaceae* species, like *Stryphnodendron adstringens* (Mart.), *Mimosa tenuiflora* (Mart.), *Mimosa arenosa* (Willd.) Poir., *Mimosa caesalpiniifolia* Benth., *Anadenanthera colubrina* var. cebil. [56], and *Plathymenia reticulata* Benth [57], are a potential source of condensed tannins. Besides this, different flavonoids have been detected in the bark of different *Fabaceae* species like quercetin, quercitrin, taxifolin, apigenin, astibilin, and kaempferol, which have been identified in the *Hymenaea martiana* bark [58]. Another study reported the presence of isoquercitrin, quercetin, and rutin in *Dimorphandra gardneriana* Tul. bark [59]. 

However, the biological properties of polyphenols depend on their bioavailability [60,61] for intestinal absorption, metabolization, and subsequent interaction with target tissues or organs [62]. In fact, the metabolism of flavonoid glycosides involves several enzymatic activities and interactions with the gut microbiota, leading to the release of bioactive aglycones [63]. For instance, glycosylation improves the solubility and bioavailability of quercetin, which can enhance its therapeutic potential as quercetin has relatively low bioavailability due to poor absorption, rapid metabolism, and extensive first-pass elimination in the liver [64].

Imidazole alkaloids have been noticed in different plant parts of some *Cynometra* species, like anantine, cynometrine, and cynodine, from *Cynometra anata* Hutch. and Dalziel (leaf) [65], N1-demethyl cynometrine, N1-demethyl cynodine, cynometrine, and cynodine from *Cynometra hankei* Harms (stem bark and seed) [66] and anantine, cynometrine, isoanantine, isocynometrine, isocynodine, noranantine, hydroxyanantine, and cynolujine from *C. lujae* (plant part not referred) [65]. However, in our study, no trace of alkaloids was detected in *C. iripa* leaf (CIL) or *C. iripa* bark extracts. 

The *C. iripa* leaf extract showed a higher total phenolic content (TPC), 1521 ± 4.71 mg of GAE/g dry weight, than the *C. iripa* bark extracts, which was 1476 ± 4.09 mg GAE/g dry weight. A higher TPC was also reported in *C. cauliflora*, in which the TPC of an aqueous extract of young leaf was 1831.47 ± 1.03 mg GAE/g [67] and a lower TPC was found in a *C. ramiflora* stem methanolic extract (96.2 mg GAE/g dry weight) exhibiting the influence of extraction methods in the quantification of the secondary metabolites in different *Cynometra* species [68].

The obtained values for the total flavonoid content (TFC) of *C. iripa* leaf and bark extracts were 64 ± 1.00 CE/g dry weight and 82 ± 0.58 mg CE/g, respectively. In a study, an aqueous extract of *C. cauliflora* leaf exhibited a TFC of 33.63 ± 0.25 mg CE/g dry weight [67], and a high TFC of 166.4 mg QE/g was reported in a methanol extract of *C. ramiflora* stem [68]. However, in our study, *C. iripa* bark extracts exhibited a higher TFC than the leaf extracts. In a study, the hydroethanolic and hydromethanolic extracts of a *Fabaceae* species named *Pongamia pinnata* (L.) Pierre bark showed a higher TFC 2.28 ± 0.01 and 3.44 ± 0.04 g CE/100 g dry weight, respectively, than the leaf extract [69]. Besides this, a higher TFC was also reported in the methanolic extract of stem bark (902 ± 0.7 mg quercetin equivalents/g) than the root and leaf extract of *Rhizophora mucronate*, which is also a mangrove species [70]. 

The total condensed tannin content (TCTC) of *C. iripa* leaf and *C. iripa* bark were 755 ± 4.4 mg and 1021 ± 5.51 mg CCE/g dry weight, respectively. A lower TCTC of 80.4 mg GAE/g dry weight was reported in another species *C. ramiflora* stem methanolic extract because of the differences in the species, plant part, extraction solvent, and methodology. In addition, we expressed our result in CCE (Cyanidin Chloride Equivalent), whereas they expressed their result in Gallic Acid Equivalent (GAE) [68]. No more studies were found in other *Cynometra* species concerning the tannin content in cyanidine chloride equivalent. 

CIL and CIB extracts showed antioxidant activity by the DPPH assay with IC_50_ 23.95 ± 0.93 and 23.63 ± 1.37 µg/mL, respectively, and by the FRAP assay with values of 61.11 ± 2.91 and 77.94 ± 2.02 µmol Fe^2+^/g dry weight, respectively. In the DPPH assay, both extracts showed a concentration-dependent scavenging activity higher than standard ascorbic acid. By the FRAP assay, both extracts showed a ferric-reducing capability lower than the used standards. Phenolic compounds, including procyanidins, were believed to be involved in the demonstrated antioxidant activity of both extracts. Comparatively, in both DPPH and FRAP assays, *C. iripa* bark (CIB) was shown to possess a higher antioxidant activity than *C. iripa* leaf (CIL). No more information has been found concerning the antioxidant activity of CIL and CIB hydroethanolic extracts. Ethanolic extracts of the leaf of another species *C. cauliflora* exhibited remarkable antioxidant activity with an IC_50_ value of 2.88 ± 0.05 µg/mL than the standard quercetin in the DPPH assay [71]. Also, aqueous extracts of fruit of the same species showed potent antioxidant capacity in both DPPH with an IC_50_ value of 0.47 ± 0.03 g of dry weight/mL and a FRAP assay with reducing power of 25.07 ± 0.73 µmol Fe^2+^/g dry weight [72]. 

A positive and statistically significant (*p* < 0.05) correlation was noticed between the phenolic content and antioxidant activity. So, these phenolic derivatives are mainly responsible for the antioxidant activity of both extracts.

## 4. Materials and Methods

### 4.1. Plant Materials 

**Figure 13:**
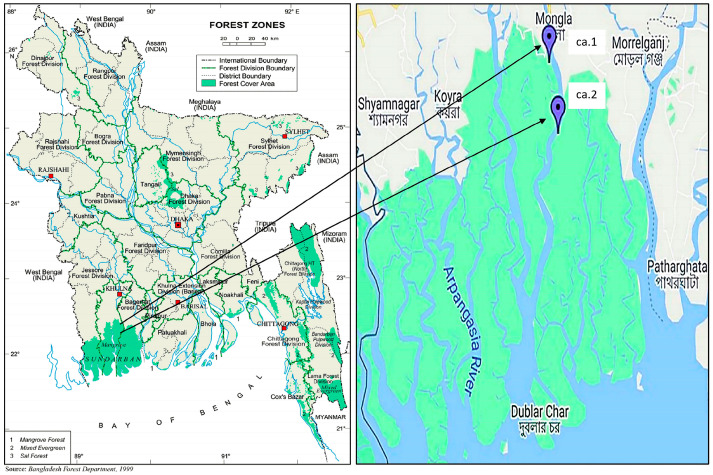
Map of Bangladesh showing plant collection area in the Sundarbans mangrove forest; ca.1—collection area 1: Koromjol and ca.2—collection area 2: Harbaria eco-tourism center, Bangladesh.

The leaf and bark of *C. iripa* were collected in April 2019 from the Koromjol and Harbaria eco-tourism center (Figure 13) in the Chadpai range of the Sundarbans, Khulna District, Bangladesh. The identification of the collected samples was confirmed by Dr. Fahmida Khanam, director, Bangladesh National Herbarium, and the corresponding voucher samples were deposited in this herbarium with the voucher number DACB-47644. A copy of them was also kept in the Laboratory of Pharmacognosy (Department of Pharmacy, Pharmacology, and Health Technologies) Faculdade de Farmacia, Universidade de Lisboa, (FFUL), Portugal. 

After identification, the plant’s raw material for laboratory studies was dried in the dark at room temperature (±22 °C) in Bangladesh and transferred to the Laboratory of Pharmacognosy at the FFUL. 

### 4.2. Botanical Studies

#### 4.2.1. Samples

To conduct the botanical analysis, fifty samples were randomly selected from the 250 g of collected raw material in accordance with the sampling guidelines set forth in the European Pharmacopoeia for herbal drugs. A representative portion of the total collected plant material was powdered using a mill and then mounted in a 60% chloral hydrate solution, following the procedures outlined in the European Pharmacopoeia [73].

#### 4.2.2. Macroscopic Analysis

The macroscopic analysis was performed with the naked eye and an Olympus SZ61 stereo microscope (Switzerland) equipped with a Leica MC170 HD digital camera. Image capture and analysis were facilitated by the Leica Application Suite (LAS) Version 4.8.0 software (Switzerland).

#### 4.2.3. Microscopic Analysis

Transverse sections (midrib, distal part of the blade, and petiolule) and tangential longitudinal sections (leaf surface) were cleared and mounted in a 60% chloral hydrate aqueous solution. Microscopic analysis of the prepared leaf sections and powdered plant material was carried out using an Olympus CX31 microscope fitted with a Leica MC170 HD digital camera, with imaging processed via the LAS Version 4.8.0 software (Switzerland).

For macroscopic feature determination, observations were made on 15 adult leaves. For microscopic measurements, 30 samples were analyzed (1 mm² per sample). The stomatal index (SI) was calculated using the following formula:

SI = (*S* × 100)/(*S* + E)

where (*S*) represents the number of stomata per unit area of the leaf and (E) the number of epidermal cells in the same area of the leaf [73]. 

### 4.3. Chemical Studies

#### Plant Extract Preparation

The hydroethanolic (70%) extracts of each herbal substance (leaf and bark) were prepared using ethanol and water in a ratio of 70:30 at room temperature by maceration (a minimum of 3 × 24 h each). This solvent mixture assures the extraction of polar and apolar secondary metabolites. After extraction and filtration using the G4 glass filter under vacuum, the solution was evaporated by a rotary evaporator (Buchi R-100, Flawil, Switzerland) at a temperature less than 40 °C and then put in the freezer (−20 °C) and finally lyophilized at −55 °C (Heto LyoLab-3000, Dietikon, Switzerland) [73]. The Drug Extract Ratio (the ratio of the amount of plant material to the amount of the obtained extract) was evaluated, and the following equation was used to calculate the percentage (%) of yield:

Yield of extraction (%, *w*/*w*) = Wt_1_/Wt_2_ × 100%

Wt_1_ and Wt_2_ represent the final weight of the dried extract and the primary weight of the leaf/bark powder [74]. 

### 4.4. Qualitative Phytochemical Analysis 

The hydroethanolic extracts were qualitatively analyzed for different secondary metabolites using conventional procedures and LC/UV-DAD/ESI-MS analysis. Preliminary phytochemical screening was conducted for alkaloids by the Bouchardat/Mayer/Dragendorff test [75], phenolic compounds by the ferric chloride test and acetic acid test [76], and saponins by the foam test [77].

#### LC-UV/DAD-ESI/MS Analysis

A Waters Alliance 2695 high-performance liquid chromatography (HPLC) system with an autosampler and photodiode array detector (Waters PDA 2996) was used in conjunction with a MicroMass Quattro MicroTM API triple quadrupole tandem mass spectrometer (Waters, Drinagh, Ireland). The separation module, also from Waters, included a quaternary pump system, degasser, autosampler, and column oven. Chromatograms were captured over a wavelength range of 210–700 nm.

An electrospray ionization source (ESI) was operated in negative mode. Separation was carried out using a LiCrospher^®^ 100 RP-18 column (5 µm, 250 × 4 mm, Merck, Darmstadt, Germany) maintained at 35 °C. The flow rate was set to 0.3 mL/min with an injection volume of 20 μL. The mobile phase comprised water containing 0.1% formic acid (Phase A) and acetonitrile (Phase B), with a total run time of 90 min. The gradient conditions were 5% Phase B at 0 min, 20% Phase B at 20 min, 50% Phase B at 60 min, and 100% Phase B at 90 min. The peaks were analyzed by MassLynx™ V4.1 software (Waters^®^, Drinagh, Ireland). The compounds were identified by co-chromatography and by comparison of retention time, UV, and mass spectral data with reference standards (quercetin-3-*O*-glucoside from Honeywell Fluka, Germany, and apigenin from Extrasynthese, Genay, France) or tentatively identified according to the literature and databases.

### 4.5. Quantitative Phytochemical Analysis

All values were obtained in 3 sets of experiments and evaluated in triplicate by spectrophotometry using a Hitachi U-2000 UV–Vis spectrophotometer (Tokyo, Japan).

- Total phenolic content

The total phenolic content of each of the extracts was determined using the Folin–Ciocalteu assay [78], where 2 mL of Folin–Ciocalteu reagent (diluted with water 1:10 *v*/*v*) was mixed with 0.4 mL of extract and then 1.6 mL of anhydrous Na_2_CO_3_ (75 g/L) solution. After two hours, the absorbance was measured at 765 nm. The gallic acid was used to obtain a standard calibration curve, and distilled water was used as blank. Results were expressed as mg of gallic acid equivalents (GAE)/g dried plant materials. Data are presented as the mean ± standard deviation.

- Total flavonoid content

The total flavonoid content of each extract was determined by using the aluminum chloride colorimetric assay by Oliveira et al. (2008) with some modifications [79]. To 0.5 mL of extract, 2 mL of distilled water and 150 µL of 5% NaNO_2_ were added, and the mixture was left to incubate for 5 min. After that, 150 µL of 10% AlCl_3_ was added and incubated for 6 min. Then, finally, 1 mL of 1M NaOH was added and incubated at 18 °C in the dark for 20 min. Absorbance was measured at 510 nm. An increasing catechin concentration was used to obtain a standard calibration curve. The results were expressed as mg of catechin equivalents (CE)/g dried plant materials. Data are presented as the mean ± standard deviation.

- Total condensed tannin content

The total condensed tannin content of each of the extracts was evaluated using the method of Porter et al. (1986) [80]. To 0.5 mL of plant extracts (diluted in 70% Acetone) we added 3 mL of butanol–HCl reagent (butanol-HCI 95:5 *v*/*v*) and 0.1 mL of ferric reagent (2% ferric ammonium sulfate in 2N HCl). Then, the solution was mixed and incubated at 97 to 100 °C for 1 h using a hot water bath. Absorbance was measured at 550 nm. The cyanidin chloride concentration was used as the standard calibration curve. The blank for each sample comprised 0.5 mL of the extract, 3 mL of butanol–HCl reagent, and 0.1 mL of the ferric reagent. Results were expressed as mg of cyanidin chloride (CCE)/g dried plant material. Data are presented as the mean ± standard deviation.

### 4.6. Antioxidant Activity 

#### 4.6.1. DPPH (2,2-Diphenyl-1-picrylhydrazyl) Free Radical Scavenging Assay

The free radical scavenging activity was determined by the DPPH assay [81]. In this assay, the purple-colored DPPH becomes reduced by a hydrogen or electron donor, and its color changes to yellow. DPPH solution (3.9 mL, 6 × 10^−5^ M in methanol) was mixed with 100 µL of each extract. After 30 min of incubation at room temperature, the absorbance of the samples and standard solution was measured at 517 nm. Ascorbic acid was used as the reference standard. The inhibition ratio (percent) was calculated from the following equation.

% Inhibition = A_0_ − A_1_/A_0_ × 100

where A_0_ = the absorbance of the control and A_1_ = the absorbance of the standard.

The IC_50_ value is the concentration of the sample required to scavenge 50% of free radicals, and we calculated this from the plot of % inhibition against the concentration of each extract.

#### 4.6.2. FRAP Assay

Under acidic conditions, the ferric 2,4,6-tri-2-pyridyl-s-triazine (Fe³⁺-TPTZ) complex is reduced to its ferrous form (Fe²⁺) by antioxidants, resulting in a vivid blue coloration with an absorption peak at 593 nm [82,83]. 

To prepare the FRAP reagent, 25 mL of acetate buffer (pH 3.6), 2.5 mL of ferric chloride solution (prepared by dissolving 0.5406 g of ferric chloride in 100 mL of distilled water), and 2.5 mL of TPTZ solution (prepared by dissolving 0.0781 g of TPTZ in 40 mM of HCl) were combined. The mixture was then incubated in a water bath at 37 °C for 10 min. For the assay, 300 μL of water and 100 μL of the test sample were added to a cuvette. About 3000 μL of the prepared FRAP reagent was subsequently introduced into the cuvette and mixed by inversion. A control assay was performed using water in place of the sample. The absorbance at 593 nm was recorded with a spectrophotometer exactly 4 min after adding the FRAP reagent. 

### 4.7. Statistical Analysis 

All the macroscopic and microscopic results were obtained by using Excel 365 software (version 2401) from Microsoft and expressed as minimum, maximum, mean ± SD, and median, except for determining the stomatal index (Table 2). Pearson’s correlation test was used to establish the correlation between TPC, TFC, TCTC, and antioxidant assays (DPPH, FRAP).

## 5. Conclusions

*C. iripa* is a medicinally important mangrove species. The different plant parts of this species are traditionally used to treat different ailments in Bangladesh and India. There are a few studies concerning the quality, safety, and efficacy of *Cynometra* species. For the first time, this study has been conducted on the establishment of quality parameters for *C. iripa* leaf and *C. iripa* bark as herbal medicines. *C. iripa* extracts have been found to be a good source of phenolic derivatives, mainly proanthocyanidins, believed to be responsible for their antioxidant activity. However, our future step will be a deeper phytochemical investigation to identify more secondary metabolites and their isolation, and besides this, pharmacological studies will be conducted to clarify their traditional use.

## Figures and Tables

**Table 1:** Previously reported chemical constituents identified in *Cynometra iripa*.

Plant Part	Chemical Class	Compounds Name	Ref.
Leaf, seed oil	Fatty acids	leaf: arachidic acid, behenic acid, *cis*-11-eicosenoic acid, lauric acid, linolenic acid, myristic acid, oleic acid, pentadecanoic acid, palmitic acid, stearic acid	[18]
		seed: arachidic acid, behenic acid, caproic acid, *cis*-8, 11, 14-eicosatrienoic acid, *cis*-13, 16-docosadienoic acid, lauric acid, lignoceric acid, linoleic acid, linolenic acid, myristic acid, oleic acid, palmitic acid, stearic acid, tricosanoic acid.	
Seed, seed coat	Terpenoids	cholesta-4,6-dien-3-beta-ol, *β*-carotene, *β*-sitosterol, stigmast-4-en-3-one, squalene	[19,20]
	Esters	1,2-benzene dicarboxylic acid mono (2-ethylhexyl) ester, butyric acid-2-pentadecyl ester, 1,2-benzene dicarboxylic acid butyl-2-ethylhexyl ester	
	Fatty alcohols	1-eicosanol, falcarinol	
	Phenols	2,5-di-*tert*-butyl-1,4-benzoquinone, 3,5-di-*tert*-butyl-4-hydroxybenzaldehyde	
	Vitamins	vitamin E	

**Table 2:** Quantitative microscopy characteristics of *C. iripa* leaf and *C. iripa* bark.

Anatomical Characteristics	Min–Max	Mean ± SD	Median
Leaf			
Total stomata dimensions (abaxial surface)			
Length (µm)	16.01–28.85	20.55 ± 2.87	19.66
Width (µm)	14.62–21.29	16.90 ± 1.83	16.36
Adaxial epidermal cell			
Length (µm)	10.26–18.64	13.53 ± 2.50	13.24
Width (µm)	5.45–13.41	8.52 ± 1.79	8.52
Abaxial epidermal cell			
Length (µm)	12.84–34.20	20.84 ± 5.97	18.86
Width (µm)	8.37–15.95	11.33 ± 1.70	11.18
Cross-sectional Features			
Total mesophyll length (µm)	108.38–164.37	132.25 ± 15.24	131.62
Xylem vessel diameter (µm)	9.19–21.21	13.50 ± 3.43	12.31
Calcium oxalate prismatic crystals width (µm)	3.53–8.70	5.89 ± 1.32	6.19
Stomatal Index	20.15 ± 3.44		
Bark			
Schlerid cell wall thickness (µm)	4.62–11.50	7.65 ± 2.15	7.13
Starch diameter (µm)	3.93–7.95	6.24 ± 1.05	6.37
Calcium oxalate prismatic crystals width (µm)	4.90–13.12	8.24 ± 1.57	7.86

Abbreviations: Min—minimum; Max—maximum; SD—standard deviation.

**Table 3:** *C. iripa* yield of extraction and drug extract ratio.

Parts Used	Weight (g)	Yield of Extract in %	DER (*m*/*m*)
CIL	200	14.9	6.71:1
CIB		17.6	5.68:1

Abbreviations: CIL—*C. iripa* leaf; CIB—*C. iripa* bark; DER—drug extract ratio.

**Table 4:** Preliminary phytochemical screening on *C. iripa* leaf and bark extracts.

Phytoconstituents	Test	Results	
		CIL	CIB
Alkaloids	Bouchardat/Mayer/Dragendorff	−	−
Phenolic compounds	Ferric chloride test	+	+
	Acetic acid test	+	+
Saponins	Foam test	+	+

Abbreviations: CIL—*C. iripa* leaf; CIB—*C. iripa* bark; + (positive); − (negative).

**Table 5:** LC-UV/DAD-ESI/MS identification of the main marker compound of *C. iripa* leaf extracts.

Plant Part	Peak	*t_R_* (min)	λ_max_ (nm)	MW	[M − H]^−^ (*m*/*z*)	Fragment Ions (*m*/*z*)	Tentative Assignment
CIL	**a**	27.77	218, 223, 279	1155	1154	865, 577, 425, 287	B-type proanthocyanidin (tetramer)
	**b**	30.39	218, 223, 279	866	865	577, 289	B-type proanthocyanidin (trimer)
	**c**	31.08	218, 223, 279	1443	1442	1154, 865, 577, 289	B-type proanthocyanidin (pentamer 1)
	**d**	31.95	218, 223, 279	1443	1442	1154, 865, 577, 289	B-type proanthocyanidin (pentamer 2)
	**e**	34.20	223, 284	436	435	303, 285, 151	taxifolin pentoside isomer 1
	**f**	36.48	223, 285	436	435	303, 285, 151	taxifolin pentoside isomer 2
	**g**	37.76	223, 268, 284, 351	464	463	435, 303, 285, 152	quercetin 3-*O*-glucoside
	**h**	38.67	223, 289	436	435	303, 285, 151	taxifolin pentoside isomer 3
	**i**	40.53	218, 268, 284, 351	434	433	301	quercetin 7-*O*-pentoside/apioside
	**j**	41.27	218, 268, 284, 347	448	447	285	kaempferol 7-*O*-glucoside
	**k**	58.85	223, 269, 334	270	269	269, 89	apigenin

Abbreviations: CIL—*C. iripa* leaf; *m*/*z*—mass to charge ratio [M − H]^−^—negative mass electrospray ionization mode; *t_R_*—retention time; λ_max_—wavelength of maximum absorbance.

**Table 6:** LC-UV/DAD-ESI/MS identification of the main marker compound of *C. iripa* bark extracts.

Plant Part	Peak	*t_R_* (min)	λ_max_ (nm)	MW	[M − H]^−^ (*m*/*z*)	Fragment Ions (*m*/*z*)	Tentative Assignment
CIB	**a′**	27.76	221, 279	1155	1154	865, 577, 425, 289	B-type proanthocyanidin (tetramer 1)
	**b′**	30.38	221, 280	866	865	577, 289	B-type proanthocyanidin (trimer 1)
	**c′**	31.08	221, 280	1155	1154	865, 577, 425, 289	B-type proanthocyanidin (tetramer 2)
	**d′**	34.78	221, 227, 280, 320	562	561	433, 287, 273	B-type proanthocyanidin (dimer 1)
	**e′**	35.32	221, 235, 280, 320	562	561	433, 287, 271	B-type proanthocyanidin (dimer 2)
	**f′**	36.47	221, 281, 346	566	565	301	quercetin-*3-O*-pentosyl-pentoside
	**g′**	37.87	221, 227, 286	450	449	303, 285, 151	taxifolin 3-*O*-rhamnoside 1
	**h′**	38.60	221, 227, 287	450	449	303, 285, 151	taxifolin 3-*O*-rhamnoside 2
	**i′**	40.18	221, 227, 284	450	449	303, 285, 151	taxifolin 3-*O*-rhamnoside 3
	**j′**	41.33	221, 228, 280, 350	600	599	447, 301	quercitrin 3″-*O*-gallate
	**k′**	58.85	223, 269, 334	270	269	269, 89	apigenin

Abbreviations: CIB—*C. iripa* bark; *m*/*z*—mass to charge ratio [M − H]^−^—negative mass electrospray ionization mode; *t_R_*—retention time; λ_max_—wavelength of maximum absorbance.

**Table 7:** Quantification of the main class of secondary metabolites of *C. iripa* leaf and bark extracts.

Plant Extracts	TPC (mg GAE/g)	TFC (mg CE/g)	TCTC (mg CCE/g)
CIL	1521 ± 4.71	64 ± 1.00	755 ± 4.00
CIB	1476 ± 4.09	82 ± 0.58	1021 ± 5.51

Abbreviations: TPC—total phenolic content; TFC—total flavonoid content; TCTC—total condensed tannin content; GAE—gallic acid equivalents; CE—catechin equivalents; CCE—cyanidine chloride equivalents; SD—standard deviation. Each value in the table is represented as mean ± SD.

**Table 8:** Correlation between *C. iripa* leaf and *C. iripa* bark phenolic content and antioxidant activity.

CIL					
Variables	TPC	TFC	TCTC	DPPH	FRAP
TPC	1				
TFC	0.96	1			
TCTC	0.97	1.00	1		
DPPH	0.85	0.96	0.94	1	
FRAP	0.92	0.99	0.99	0.99	1
**CIB**					
Variables	TPC	TFC	TCTC	DPPH	FRAP
TPC	1				
TFC	0.99	1			
TCTC	0.99	1	1		
DPPH	1.00	0.99	0.99	1	
FRAP	0.95	0.99	0.99	0.96	1

Abbreviations: CIL—*C. iripa* leaf; CIB—*C. iripa* bark; TPC—total phenolic content; TFC—total flavonoid content; TCTC—total condensed tannin content; DPPH—2,2-Diphenyl-1-picrylhydrazyl; FRAP—ferric-reducing antioxidant power.

## Data Availability

The original contributions presented in the study are included in the article, further inquiries can be directed to the corresponding authors.
